# *Fgf8a* mutation affects craniofacial development and skeletal gene expression in zebrafish larvae

**DOI:** 10.1242/bio.039834

**Published:** 2019-08-30

**Authors:** I. G. E. Gebuijs, S. T. Raterman, J. R. Metz, L. Swanenberg, J. Zethof, R. Van den Bos, C. E. L. Carels, F. A. D. T. G. Wagener, J. W. Von den Hoff

**Affiliations:** 1 Department of Orthodontics and Craniofacial Biology, Radboudumc, Nijmegen, The Netherlands; 2 Department of Orthodontics and Craniofacial Biology, Radboud Institute of Molecular Life Sciences, Nijmegen, The Netherlands; 3 Department of Animal Ecology and Physiology, Radboud University, Nijmegen, The Netherlands; 4 Department of Human Genetics, Radboudumc, Nijmegen, The Netherlands; 5 Department of Oral Health Sciences and Department of Human Genetics, KU Leuven, Leuven, Belgium

**Keywords:** Zebrafish, FGF8, Craniofacial development, Morphology, Gene expression, Bone, Cartilage

## Abstract

Craniofacial development is tightly regulated and therefore highly vulnerable to disturbance by genetic and environmental factors. Fibroblast growth factors (FGFs) direct migration, proliferation and survival of cranial neural crest cells (CNCCs) forming the human face. In this study, we analyzed bone and cartilage formation in the head of five dpf *fgf8a^ti282^* zebrafish larvae and assessed gene expression levels for 11 genes involved in these processes. In addition, *in situ* hybridization was performed on 8 and 24 hours post fertilization (hpf) larvae (*fgf8a*, *dlx2a*, *runx2a*, *col2a1a*). A significant size reduction of eight out of nine craniofacial cartilage structures was found in homozygous mutant (6–36%, *P*<0.01) and heterozygous (7–24%, *P*<0.01) larvae. Also, nine mineralized structures were not observed in all or part of the homozygous (0–71%, *P*<0.0001) and heterozygous (33–100%, *P*<0.0001) larvae. In homozygote mutants, *runx2a* and *sp7* expression was upregulated compared to wild type, presumably to compensate for the reduced bone formation. Decreased *col9a1b* expression may compromise cartilage formation. Upregulated *dlx2a* in homozygotes indicates impaired CNCC function. *Dlx2a* expression was reduced in the first and second stream of CNCCs in homozygous mutants at 24 hpf, as shown by *in situ* hybridization. This indicates an impairment of CNCC migration and survival by *fgf8* mutation.

## Abstract (precis)

**Summary:** A function-blocking mutation in fgf8a causes craniofacial malformations in zebrafish larvae due to impaired cranial neural crest cell migration and survival.

## INTRODUCTION

In the human embryo, craniofacial development starts around week 4 with the formation of five facial prominences in the pharyngeal arches by the differentiation of cranial neural crest cells (CNCCs) into chondroblasts (Sperber et al., 2010; Hall and Hörstadius, 1989). These prominences give rise to the different parts of the face, including the mandible, maxilla, palate, lips and nose. Some parts of the adult craniofacial skeleton are formed by endochondral ossification that involves the replacement of a cartilage template by bone (Ornitz and Marie, 2015). Other bones such as the skull bones, mandibular body, maxilla and palate, are formed by intramembranous ossification, which is the direct deposition of bone by osteoblasts that may be also neural crest cell-derived (Ornitz and Marie, 2015). The formation of the bony and cartilaginous elements involved in morphogenesis of the mammalian head is tightly controlled by a network of signaling pathways, including bone morphogenetic proteins (BMPs), fibroblast growth factors (FGFs), Wingless-int (Wnt) and Hedgehog (Hh) proteins (Salazar et al., 2016; Litsiou et al., 2005; St-Jacques et al., 1999; Monroe et al., 2012; Ornitz and Marie, 2015; Abzhanov et al., 2007). FGFs and their receptors seem to be crucial in the initial phase of craniofacial development as they direct the migration, proliferation and survival of CNCCs that form the pharyngeal arches (Crump et al., 2004; Creuzet et al., 2004). Next to that, FGFs are also crucial in the development of the lip, palate and teeth (Stanier and Pauws, 2012; Nie et al., 2006).

In humans, 22 fibroblast growth factors (FGF1-14, 16-23) can bind to and activate four distinct fibroblast growth factor receptors (FGFR1-4) (Teven et al., 2014; Ornitz and Marie, 2015). The FGFs function as autocrine, paracrine or endocrine factors. Upon binding of FGFs to their cognate receptor, specific tyrosine residues are phosphorylated leading to activation of four different intracellular signaling cascades: the RAS-MAPK, PI3K, PLCγ and STAT pathways (Ornitz and Itoh, 2015). FGF family members have key functions in early development, in particular in cell differentiation and survival and during pattern formation. Also, FGFs are essential regulators of skeletal development as they act as chemokines by recruiting and activating chondroblasts and osteoblasts (Ornitz and Marie, 2015; Horton and Degnin, 2009; Marie, 2012).

An important player in craniofacial development is FGF8 as it is involved in CNCC migration, differentiation and survival, and development of the pharyngeal arches and the palate (Trumpp et al., 1999; Kubota and Ito, 2000; Abu-Issa et al., 2002; Shao et al., 2015). In mice, *Fgf8* is specifically expressed at the site of fusion of the medial and lateral nasal prominences that form the lip and primary palate (Bachler and Neübuser, 2001; Wilke et al., 1997). Mice with a heterozygous *Fgf8* null allele display the craniofacial phenotype of the human 22q11 syndrome with an underdeveloped jaw and cleft of the bony palate (Frank et al., 2002). In both humans and mice, mutations in the *Fgf8* gene cause deficiency of the gonadotropin-releasing hormone, leading to idiopathic hypogonadotropic hypogonadism (IHH) with or without a defect in the sense of smell (Kallmann syndrome and normosmic IHH, respectively) (Falardeau et al., 2008). In that same study, six human IHH patients carrying different point mutations in conserved residues of the *Fgf8* gene were identified, three of which also had a cleft in the lip and/or palate (Falardeau et al., 2008). In a DNA sequencing study, an individual with bilateral cleft lip and palate was identified with a *de novo FGF8* mutation (Riley et al., 2007). Structural analysis showed that this mutation causes a conformational change in the FGF8 protein that reduces the binding affinity to its cognate receptors resulting in a loss of function.

Several studies using zebrafish (*Danio rerio*) as a model system have investigated genes involved in craniofacial development and provided new clues on the etiology of human syndromic and non-syndromic cleft lip and/or palate (CL/P), as reviewed recently (Duncan et al., 2017). For example, variants of IRF6, a known regulator of human palatogenesis, are implicated both in syndromic (Van der Woude syndrome) and non-syndromic CL/P (Kondo et al., 2002). In zebrafish, *irf6* dominant-negative mutants show a cleft of the ethmoid plate (a model of the mammalian hard palate; Mork and Crump, 2015), caused by impaired neural crest cell migration (Dougherty et al., 2013). As the *irf6* gene is highly conserved, mutations in amniotes may also disrupt CNCC migration and integration during palatogenesis, eventually leading to CL/P (Dougherty et al., 2013). Alternatively, some patients with Van der Woude syndrome have a mutation in the *GRHL3* gene, a downstream target of IRF6 (Peyrard-Janvid et al., 2014). Zebrafish research revealed a key role for this gene in oral periderm differentiation, which is indispensable for human palatogenesis (de la Garza et al., 2013).

**Fig. 1.**
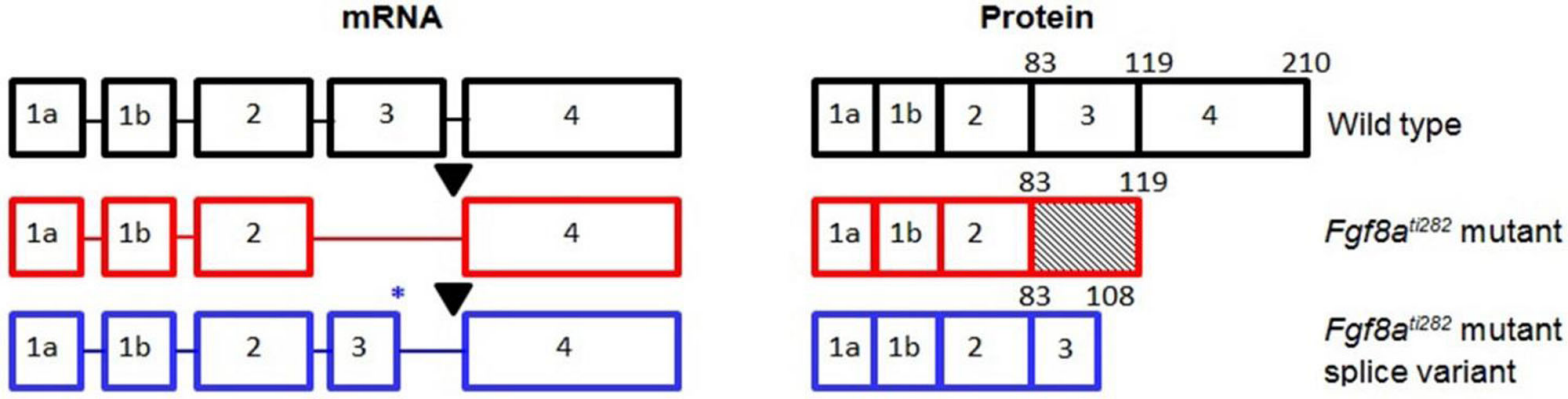
**Schematic representation of wild-type zebrafish Fgf8a mRNA and protein (in black), and possible variants caused by the *fgf8a*^ti282^ mutation (in red and blue).** The *fgf8a* gene consists of five exons. As a result of a point mutation at the 5′ splice donor site following exon 3 (black triangle), the *fgf8a^ti282^* mutant mRNA lacks exon 3 and the reading frame runs into a stop codon, resulting in a truncated, non-functional protein (Reifers et al., 1998). In some mutants, an alternatively spliced variant of the mutant allele was found, in which we revealed that part of exon 3 was retained through the use of an alternative splice donor site (blue asterisk). This variant also translates into a truncated, presumably non-functional protein (Draper et al., 2001). The gene and protein structures are not drawn to scale. The numbers above the protein structures indicate the amino acids.

In the present study we address the role of Fgf8 in craniofacial development using the zebrafish mutant line *fgf8a^ti^*^282^ (also reported as acerebellar mutant, *ace*; Reifers et al., 1998). The mutant allele has a point mutation (G>A at position 282) in the splice donor site following exon 3, which leads to skipping of exon 3 and the reading frame to run into a stop codon. The resulting truncated protein is non-functional since it lacks the motif required for receptor activation (Reifers et al., 1998) (Fig. 1).

Previous studies using homozygous *fgf8a^ti282^* knockout zebrafish larvae at 4 and 5 days post fertilization (dpf) have reported impaired craniofacial development, showing incompletely formed or even entirely lost cartilage elements (Crump et al., 2004; Choe and Crump, 2014). These studies have only briefly described the *fgf8a^ti282^* mutant craniofacial phenotype, mainly focusing on the lower jaw region. Up to now, an extensive analysis of the effects of the lack of Fgf8a on craniofacial development is lacking. Also, effects of the mutation on downstream signaling pathways in bone and cartilage formation have not been assessed yet. In the current study, a morphometric analysis of nine cartilaginous and nine mineralized elements in the zebrafish head was performed on 5 dpf *fgf8a^ti282^* homo- and heterozygous larvae, which was compared to wild type. In addition, expression of 11 genes crucial in bone and cartilage formation was assessed in the three groups. Also, *in situ* hybridization was performed for selected genes at 8 and 24 hours post fertilization (hpf). We believe this research contributes to the understanding of the role of FGF8 in craniofacial development in vertebrates.

## RESULTS

**Fig. 2.**
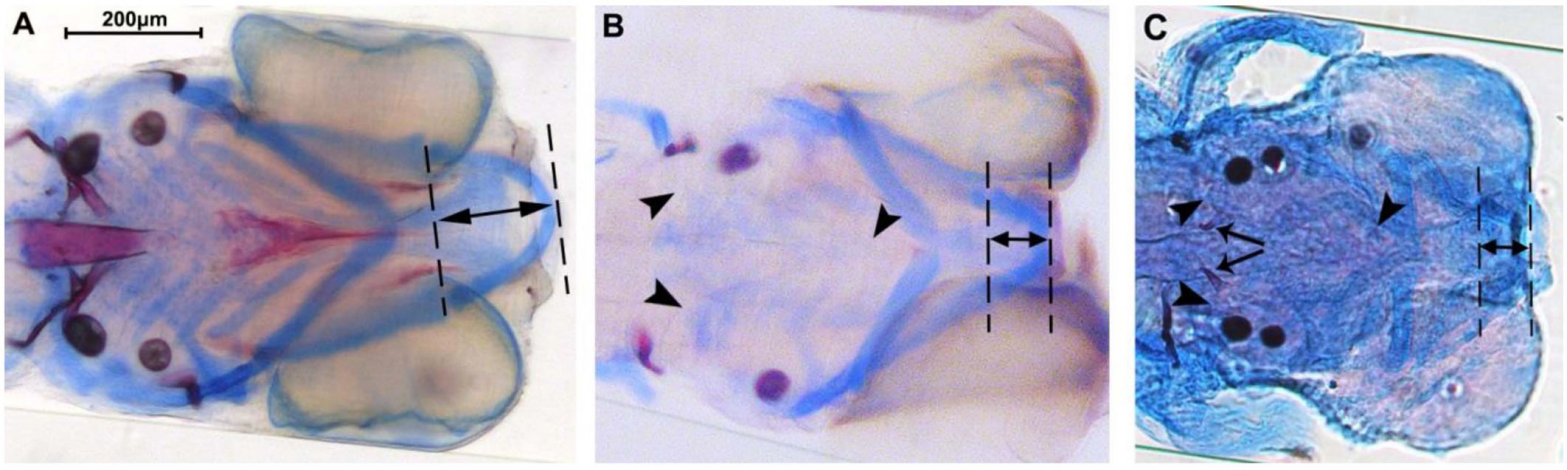
**Representative examples of a 5 dpf wild-type (A), *fgf8a*^ti282^ heterozygous (B) and -homozygous (C) larvae.** Larvae were stained for cartilage (blue) and bone (red). Note the difference in head size; scale bar: 200 µm. Also note the absence of mineralization, such as ceratobranchial 5 and the parasphenoid bone in the hetero- and homozygote (arrowheads), and the presence of teeth in the homozygote (arrows). Note the considerably smaller Meckel's cartilage in hetero- and homozygotes (double-headed arrows).

Wild-type (*n*=49), *fgf8a^ti282^* heterozygous (*n*=43) and *fgf8a^ti282^* homozygous (*n*=31) larvae were stained for cartilage and mineralized tissue. *Fgf8a^ti282^* homo- and heterozygous larvae showed a comparable reduction in size of the head, which seemed to affect mainly the anterior part. It was also clearly visible that a number of cartilage structures were underdeveloped and bone structures were less mineralized in homo- and heterozygous larvae (Fig. 2).

### Aberrant cartilage phenotype in *fgf8a^ti282^* homo- and heterozygous larvae

**Fig. 3.**
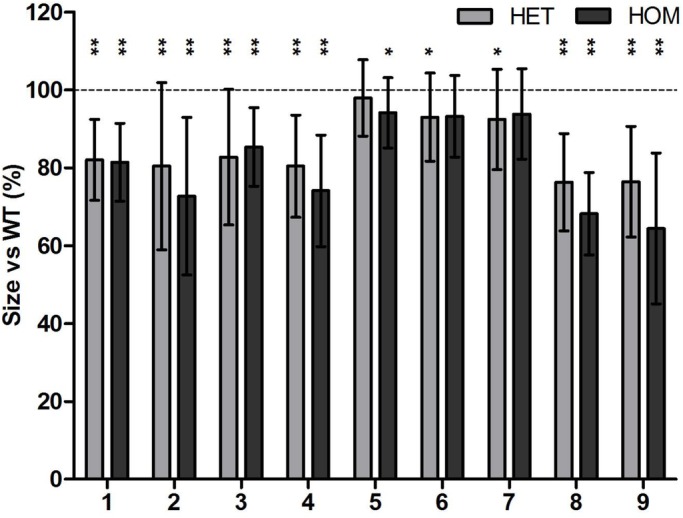
**Morphometrical analysis of nine different cartilage elements in 5** **dpf *fgf8a*^ti282^ homozygous and heterozygous mutants in comparison to wild type.** On the vertical axis the reduction in size is expressed as percentage of the wild type (set at 100%). Parameters are: (1) total head length, (2) length ceratohyal to anterior end of the head, (3) length ceratohyal to posterior end of the head, (4) width at Meckel's cartilage and palatoquadrate joint, (5) width at ceratohyal and palatoquadrate joint, (6) length of superior ceratohyal, (7) length of inferior ceratohyal, (8) length of ethmoid plate, (9) lateral length of Meckel's cartilage. Error bars represent standard deviations. Asterisks represent significant difference compared with wild type (*=*P*<0.05, **=*P*<0.01, *N*=30 or more).

Eight (numbers one to four, six to nine) out of nine parameters were found to be significantly smaller in size for *fgf8a^ti282^* homo- and heterozygotes compared to wild type (Fig. 3). Only parameter number five, the width between the ceratohyal-palatoquadrate joints on both sides, was not significantly different for both genotypes. The largest reduction in size in both homo- and heterozygotes was found for the length of the ethmoid plate (32 and 24% lower than in wild type) and the length of the Meckel's cartilage (36 and 24%, respectively). First arch structures, such as Meckel's cartilage (four, nine) and the ethmoid plate (eight), showed a much larger reduction compared to those of the superior and inferior ceratohyals, both derived from the second arch. A slightly smaller, but significant size reduction was found for the length of the anterior and posterior parts of the head, and the total head length (parameters one, two and three). Also, the width anteriorly in the head (parameter four) was significantly reduced, whereas the width more posteriorly (parameter five) was not significantly reduced, corresponding with the regions derived from respectively pharyngeal arch 1 and 2.

### Reduced bone formation in *fgf8a^ti282^* homo- and heterozygous zebrafish

**Fig. 4.**
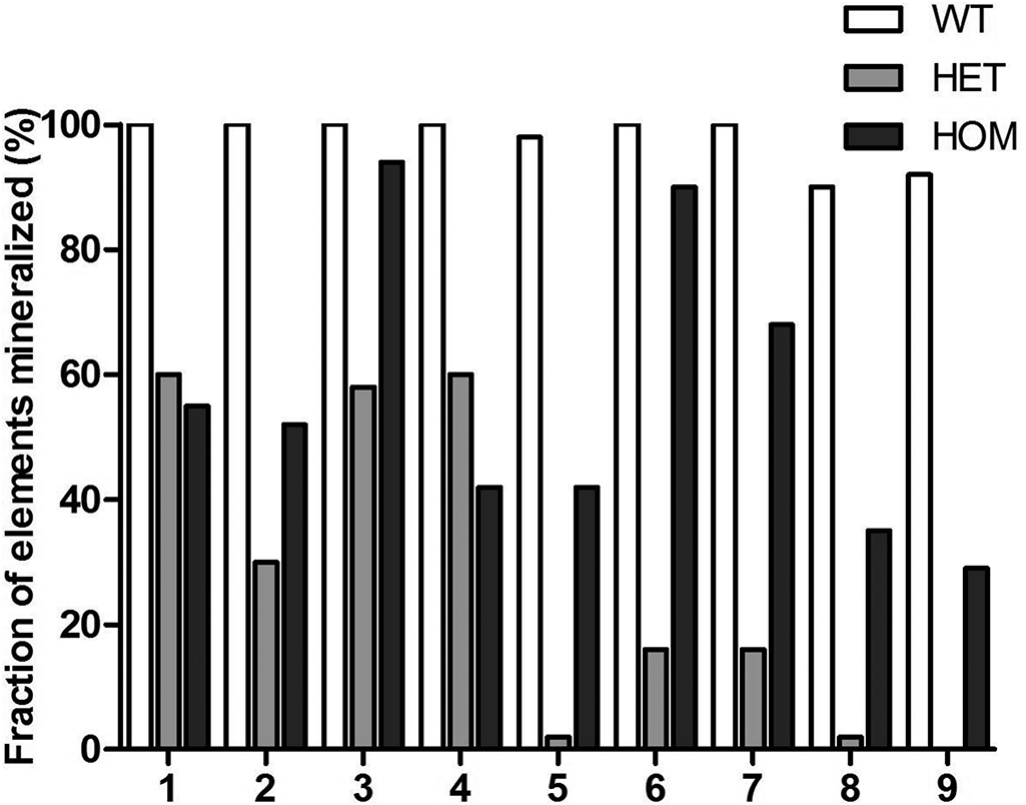
**Presence of nine different mineralized craniofacial (bone) elements in 5** **dpf mutants, heterozygotes and wild type.** On the vertical axis, the presence of the nine mineralized structures is expressed in the number of individuals studied. Parameters scored were: (1) parasphenoid, (2) otoliths (four in total), (3) notochord, (4) cleithrum, (5) ceratobranchial 5, (6) teeth (on ceratobranchial 5), (7) opercles, (8) branchiostegal ray 1, (9) entopterygoid bone. For all elements the three groups were significantly different from wild type (*P*<0.001).

In 17 out of 43 (40%) of the *fgf8a^ti282^* heterozygous larvae and 14 out of 31 (45%) of the homozygous larvae, the parasphenoid bone (one of the first bone structures that ossifies) was not mineralized, while it was mineralized in all wild-type larvae. Also, eight other mineralized structures were often absent in homo- and heterozygous larvae, with heterozygous larvae being mostly affected (Fig. 4). For instance, ceratobranchial 5 and branchiostegal ray 1 were not mineralized in 98% of the heterozygous larvae, while both structures were found in almost all wild-type larvae (98% and 90%, respectively). In homozygotes, ceratobranchial 5 was not mineralized in 58% of the larvae, although often mineralized teeth were already present. The homozygotes never showed a complete loss of an element. The presence of mineralized structures was significantly different between the three groups for all parameters (*P*<0.001).

### Altered gene expression in homozygous *fgf8a^ti282^* larvae

**Fig. 5.**
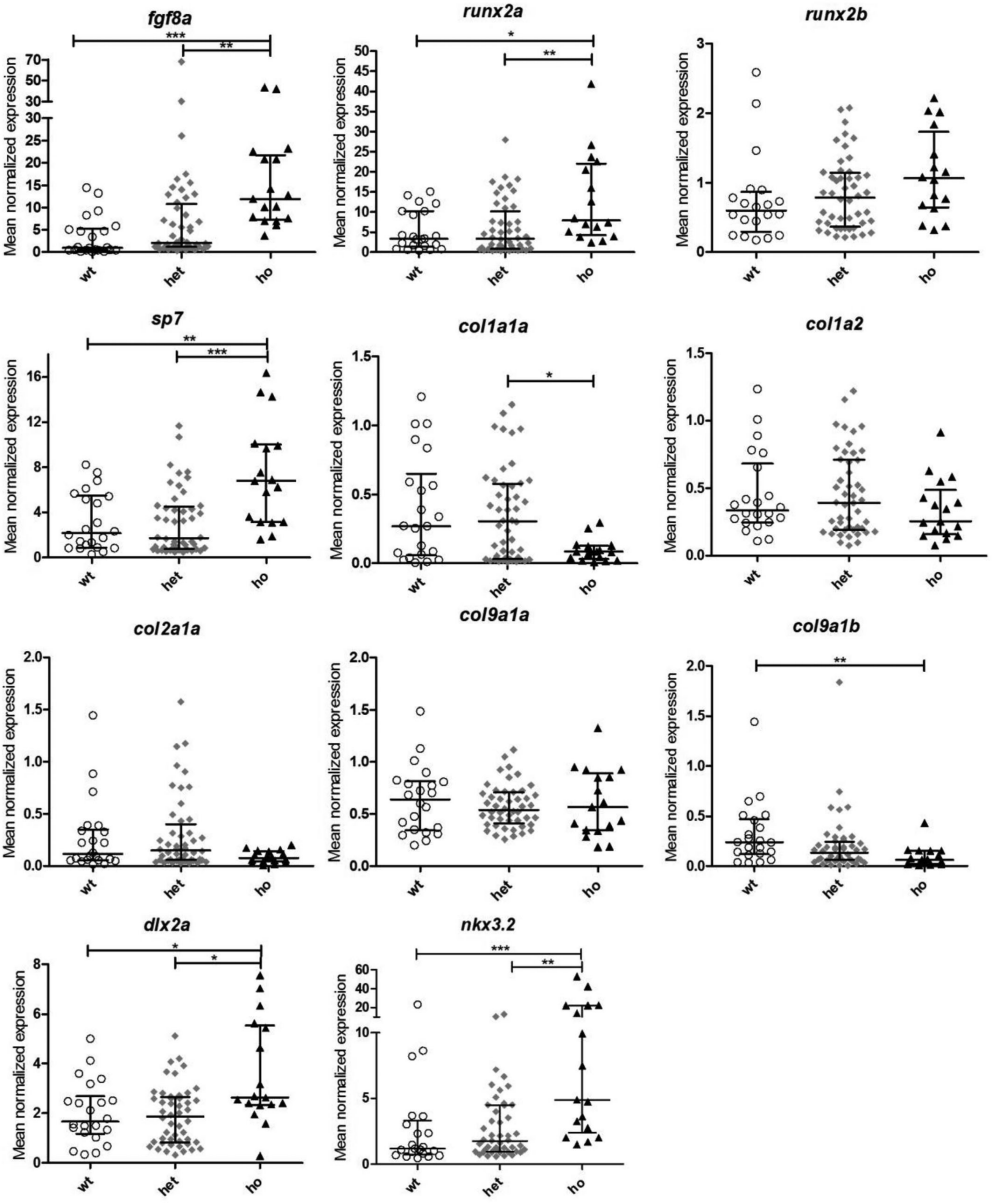
**Relative expression levels for 11 genes in 5** **dpf wild-type, heterozygote and mutant *fgf8a*^ti282^ zebrafish.** Each data point represents one individual; the horizontal line depicts the median, error bars indicate the interquartile ranges. The expression was assessed for *fgf8a* (A), *runx2a* (B), *runx2b* (C), *sp7* (D), *col1a1a* (E), *col1a2* (F), *col2a1a* (G), *col9a1a* (H), *col9a1b* (I), *dlx2a* (J) and *nkx3.2* (K). Asterisks indicate the significance level: *=*P*<0.05, **=*P*<0.01, ***=*P*<0.001.

To shed light on the observed changes in formation of cartilage and bone elements, the expression of 11 different genes, related to bone and cartilage, and neural crest cells was evaluated (Fig. 5). The groups consisted of *fgf8a^ti282^* homozygous mutant (*n*=17), heterozygous (*n*=49) and wild-type (*n*=22) larvae. The homozygous mutants showed a significant upregulation of *fgf8a* compared to wild type (*P*<0.001) and heterozygotes (*P*<0.01). The expression of the osteoblast genes *runx2a* (an essential transcription factor early in osteoblast differentiation) and *sp7* (a transcription factor that is crucial for differentiation of preosteoblasts to mature osteoblasts, as well as to increase osteoblast activity), was significantly upregulated in the mutant larvae compared to both the wild-type and heterozygous larvae. In contrast, the bone matrix gene *col1a1a* was significantly downregulated in mutants; *col1a2* did not show any differences between the three groups. *Col2a1*, which encodes a chain of collagen type II, an extracellular matrix (ECM) component of cartilage, showed a reduced expression level in mutants (Fig. 5). One of the genes also encoding a component of hyaline cartilage, *col9a1b*, was found to be significantly lower expressed in homozygotes compared to the wild type (72%, *P*<0.01), although expression of the related *col9a1a* was not affected. The CNCC marker gene *dlx2a* was significantly upregulated in the homozygous mutants compared to both wild type and heterozygotes (*P*<0.05). Also the gene encoding NK homeobox 2 (*nkx3.2* or *bapx1*), involved in joint patterning, was upregulated in mutants compared to wild type (*P*<0.001) and heterozygotes (*P*<0.01). In contrast, the heterozygous larvae did not show any significant differences in gene expression compared with the wild type.

### Spatiotemporal gene expression in fgf8a^ti282^ mutants during early development

**Fig. 6.**
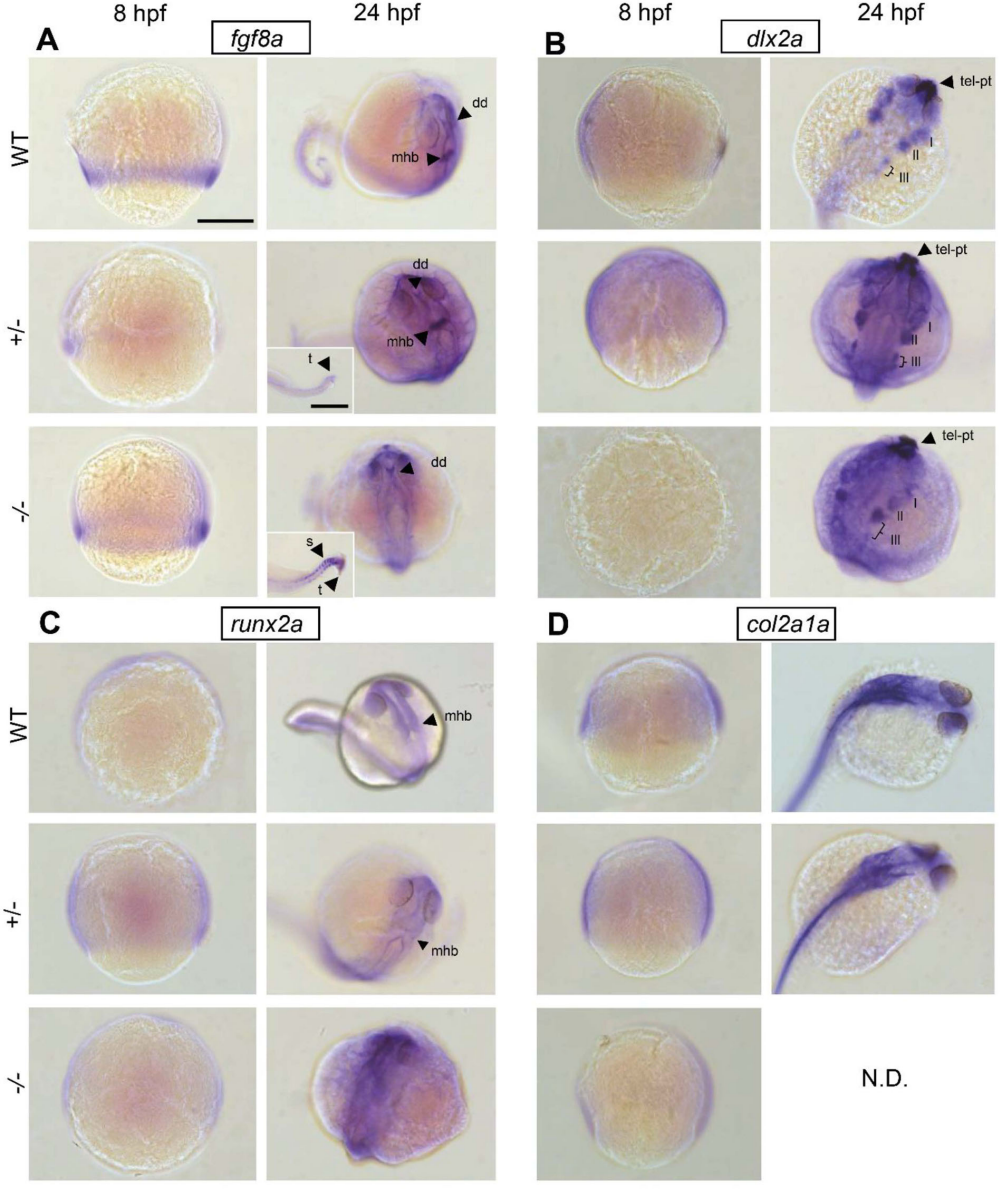
**Early spatiotemporal gene expression in** ***ace*** **mutant development.** Wholemount *in situ* hybridization in 8 hpf and 24 hpf wild-type, heterozygous and homozygous embryos shows patterns of early gene expression of (A) *fgf8a*, (B) *dlx2a*, (C) *runx2a* and (D) *col2a1a*. mhb, mid-hindbrain boundary; dd, dorsal diencephalon; t, tail end; s, somites; pt, prethalamus; tel, telencephalon; fb, forebrain; hb, hindbrain. N.D.: no data were obtained. Scale bar: 200 µm, in insert: 50 µm.

Wholemount *in situ* hybridization showed that *fgf8a* is expressed towards the vegetal pole at 8 hpf in homozygous, heterozygotes and wild-type embryos (Fig. 6A). At 24 hpf *fgf8a* was highly expressed in the dorsal diencephalon and the mid-hindbrain boundary in heterozygotes and wild-type larvae, while the structure was absent in the homozygous mutant larvae. Additionally, we showed abundant *fgf8a* expression in the posterior somites and tail end of homozygous mutant compared to heterozygous larvae.

The homeobox transcription factor *dlx2a* was not detected in 8 hpf homozygous mutant embryos but only in heterozygous and wild-type embryos (Fig. 6B). At 24 hpf *dlx2a* expression defines a subpopulation of CNCCs precursors for the pharyngeal arches. These distinct populations migrate laterally and will form the craniofacial bone and cartilage elements. In homozygous, heterozygous and wild-type larvae four well-separated populations of CNCCs were distinguished bilaterally. Our data show that populations I and II expressed more *dlx2a* in wild-type and heterozygous larvae, whereas in homozygous mutant larvae expression was reduced in these populations and most abundant in the third (III) population.

We noted a slightly increased expression of *runx2a* in 8 hpf heterozygous and homozygous mutant embryos (Fig. 6C). Moreover, at 24 hpf *runx2a* was expressed throughout the cranial region with an increased intensity in the homozygous mutant larvae.

At 8 hpf the *col2a1a* expression was slightly reduced in the mutant embryos as compared to wild-type and heterozygous embryos (Fig. 6D). *Col2a1a* positive cells in the postchordal area seemed to be located more anteriorly in the wild-type versus the heterozygous larvae at 24 hpf.

## DISCUSSION

We analyzed the effects of the *fgf8a^ti282^* mutation on bone and cartilage formation, related gene expression in 5 dpf zebrafish larvae. Homo- and heterozygous *fgf8a^ti282^* zebrafish larvae were compared to wild-type littermates for differences in bone and cartilage development, the expression levels of 11 developmental genes. In addition, *in situ* hybridization was performed for selected genes at 8 and 24 hpf. Our data show that bone and cartilage formation is severely impaired in both *fgf8a^ti282^* homo- and heterozygous larvae in comparison to wild type. *Fgf8a^ti282^* homozygous mutant larvae also show differences in expression levels of bone, cartilage and CNCC marker genes.

All evaluated mineralized structures were significantly less identified, or even completely absent in *fgf8a^ti282^* heterozygous larvae compared to wild-type siblings. In these larvae, often the opercles and branchiostegal rays could not be identified, and all of the larvae did not show any mineralization of the entopterygoid bone. Interestingly, in 40% of the heterozygous larvae the parasphenoid bone – an intramembranous bone that is part of the roof of the mouth – was also not observed. In our study, the homozygous mutant larvae showed similar results as the heterozygotes, with absence of mineralization of certain elements. The lowest presence of mineralization was seen for the branchiostegal rays (35%) and entopterygoid bone (29%). In a previous study, nearly all *fgf8a^ti282^* homozygous mutant zebrafish larvae were also missing the branchiostegal rays (Albertson and Yelick, 2007). Interestingly, the homozygotes often had teeth, although ceratobranchial five was not mineralized, which has been reported earlier (Crump et al., 2004). A study in *Fgf8* knockout mice shows that newborns are also missing skeletal elements, specifically those derived from the first pharyngeal arch, such as Meckel's cartilage and also some of the second arch (Trumpp et al., 1999). The missing elements included the palatine and pterygoid bones, parts of the roof of the mouth, which we take as similar to our findings in zebrafish larvae. These *Fgf8* mouse mutants also show features reminiscent of agnathia and holoprosencephaly, which are also found in patients with first arch syndromes (Bixler et al., 1985). Therefore, it seems that disturbed FGF8 signaling in both zebrafish and mice mainly affects the development of bones derived from the first pharyngeal arch.

In addition to the bone defects, the *fgf8a^ti282^* homo- and heterozygous mutant larvae also showed multiple deformed cartilage elements. Again, the largest deformations were seen in cartilage structures derived from the first pharyngeal arch, which corroborates the bone findings. The ethmoid plate and Meckel's cartilage were the most affected structures. Second arch structures and those of mixed origin were affected to a lesser extent. A previous study on 4 dpf *fgf8a* mutant larvae also reported craniofacial defects in Meckel's cartilage, the ethmoid plate and the ceratohyals (Crump et al., 2004), but these were not quantified. Mouse embryos carrying one null allele and one hypomorphic allele for *Fgf8* (*Fgf8^neo/−^*) showed defects in Meckel's cartilage and the bones of the palate at stage E18.5 (Abu-Issa et al., 2002). Altogether, this indicates a crucial role for Fgf8 in the development of mainly first arch-derived bone and cartilage structures. In the more posterior arches, Fgf3 might partially compensate for the loss of Fgf8 (Walshe and Mason, 2003).

To clarify the downstream effects of the *fgf8a* mutation, the expression levels of 11 genes related to bone, cartilage and CNCCs were analyzed. The osteoblast transcription factors *runx2a* and *sp7* were upregulated only in the homozygous mutant larvae compared to the wild type*. Runx2* is an essential transcription factor for early osteoblast differentiation, while *sp7* is mainly active during maturation of osteoblasts (Li et al., 2009). Zebrafish have two orthologous genes of *Runx2*: *runx2a* and *runx2b*. The upregulation of *runx2a* and *sp7* in the homozygote *fgf8a* zebrafish mutants might be caused by the loss of inhibition of the BMP-2 induced expression of Runx2 and SP7. BMP-2 is normally downregulated by FGF8 (Katsuyama et al., 2015). Also, in the *in situ* hybridization *fgf8* expression seemed to be enhanced already at 24 hpf. This corresponds with a mouse study showing that downregulation of genes like *Runx2* and *Sp7* occurred when FGF8 signaling was locally enhanced (Xu et al., 2018). Presumably, this response acts as a mechanism to compensate for the reduced bone formation. *Runx2* and *Sp7* regulate the expression of bone matrix genes including *Coll1a1* and *Coll1a2* (Komori, 2010; Koga et al., 2005). Overexpression of *Runx2* is known to inhibit osteoblast differentiation and reduce *Col1a1* expression (Komori, 2010). In our study, we also found that the expression of both bone matrix genes was lower in homozygous mutants although only *col1a1a* was significantly reduced. Normally, expression of these bone matrix genes increases during maturation of osteoblasts (Li et al., 2009). Together with the upregulated osteoblast transcription factors, this indicates that the maturation of osteoblasts is inhibited in homozygous *Fgf8a^ti282a^* larvae.

In the heterozygotes and the wild type, the expression levels of *col1a1a* and *col1a2* were unchanged. Also for the other bone-related genes, the heterozygous larvae showed no differences in expression compared to wild type. However, they did show reduced bone formation in the morphometric analysis. Since we used whole larvae for expression analysis, local effects in the craniofacial area might have been obscured.

The expression of *col9a1a* was similar in all three genotype groups, while *col9a1b* was significantly reduced in homozygotes. These genes encode two types of α-chains of type IX collagen, a minor component of hyaline cartilage. If any of these two genes is mutated, it leads to bone dysplasia in humans (Briggs et al., 1994) and a weaker ECM with disintegrated collagen II fibers in zebrafish (Huang et al., 2009). *Col2a1a*, encoding a component of collagen II, found in cartilage (Yan et al., 1995) did not show a difference in expression between the three groups. This indicates that chondrocytes are present but the cartilage might be weaker in homozygous mutants because of a lack of *col9a1b*. The reduction in size of the cartilage elements might also be due to a reduced survival of chondrocytes in both hetero- and homozygotes, which was reported in a mouse study (Abu-Issa et al., 2002).

The expression of *fgf8a* in homozygote mutants at 5 dpf is significantly upregulated compared to the wild type and the heterozygotes. *In situ* hybridization for *fgf8a* at 24 hpf clearly showed that the mid-hindbrain barrier is missing in homozygotes. As the *fgf8a^ti282^* mutant alleles produce a non-functional protein (Reifers et al., 1998), a feedback loop might be activated to compensate for the lack of functional Fgf8 protein. Apparently, this feedback loop is not activated in heterozygotes.

We furthermore showed that the expression of *dlx2a* and *nkx3.2*, two genes that are known to be inhibited by Fgf8 (Walshe and Mason, 2003; Wilson and Tucker, 2004), is enhanced in homozygote mutants. *Dlx2a* is involved in tooth development and is expressed in migrating CNCCs that contribute to the pharyngeal arches (Sperber et al., 2008), while *nkx3.2* is involved in jaw joint formation (Miller et al., 2003). *Dlx2a* is expressed by a subset of premigratory, migratory and condensating cranial neural crest cells (Yan et al., 2005). During the 23-somite stage three cell streams on both sides of the developing brain express *dlx2a.* The first stream will form the mandibular arch structures, the second stream of cells forms the structures of the hyoid arch, and the third stream separates subsequently into five distinct cell groups that will form the ceratobranchial cartilages (gill arch 3–7) (Mork and Crump, 2015). *In situ* hybridization of *dlx2a* at 24 hpf showed that the third cell stream has started to form the fourth population of CNCCs. Interestingly, we observed a decrease in *dlx2a* in the first and second stream in homozygous mutant larvae as compared to wild type and heterozygotes. This supports the morphological defects in first and second arch structures at 5 dpf.

It is also known that FGF8 inhibits the expression of *Dlx2* in mice (Thomas et al., 2000) and that of *nkx3.2* in zebrafish (Wilson and Tucker, 2004). Therefore, it seems that the upregulation of these two genes is caused by the loss of inhibition by Fgf8. The upregulation of *dlx2a* in the homozygotes might explain the presence of teeth, as it stimulates the development of the pharyngeal dentition (Borday-Birraux et al., 2006). Heterozygous *fgf8a^ti282^* mutants showed no upregulation for *dlx2a* and *nkx3.2*. Yet, the morphometric analysis showed that cartilage formation is also affected in heterozygotes. This indicates that cartilage formation is impaired by other downstream effects of the mutation.

Altogether, the data suggest that a lack of functional Fgf8 impairs CNCC migration into the pharyngeal arches, their differentiation into osteoblasts and chondroblasts, and subsequent bone and cartilage formation. *Fgf8^neo/−^* mutant mouse embryos, carrying one null allele and one hypomorphic allele for *Fgf8*, showed increased cell death in migrating CNCCs (Abu-Issa et al., 2002). In fact, our homo- and heterozygous mutant zebrafish are anticipated to also have lower levels of functional Fgf8, which might induce CNCC death and reduced cartilage formation.

In our genotype analysis on cDNA, some of the *fgf8a^ti282^* homozygote mutants were found to express an alternatively spliced mutant allele. The mutant allele carries a point mutation (G>A at position 282) that disables the splice site following exon 3 (Fig. 1). The splice variant results from the use of a cryptic splice donor site located 32 bases upstream of the original splice site (Draper et al., 2001). It is unknown how the use of the cryptic donor splice site is regulated, but translation leads to a premature stop codon and a truncated, presumably non-functional Fgf8.

## CONCLUSION

In conclusion, bone and cartilage formation is impaired in *fgf8a^ti282^* homo- and heterozygous mutant larvae. As mainly first arch structures are affected, this points towards the impairment of CNCC function, which is supported by the gene expression and *in situ* hybridization data. Both the zebrafish neurocranium and the human craniofacial region are derived from migrating CNCCs. It appears that a conserved genetic network including *fgf8a* regulates the formation of this region in all vertebrates (Mork and Crump, 2015; Swartz et al., 2011). We have shown that a mutation in *fgf8a* also influences the expression of genes regulating bone and cartilage formation such as *runx2a*, *sp7* and *col1a1a*, as well as *dlx2a*, a gene involved in the migration of CNCCs. As *fgf8a* is highly conserved from fish to mammals, mutations in the *FGF8* gene in humans may also have negative effects on CNCCs and skeletogenesis. These mutations may lead to craniofacial malformations such as CL/P. In a human genetic study on non-syndromic cleft lip and palate, an individual with bilateral CL/P was found carrying a missense mutation in the *FGF8* gene (Riley et al., 2007). This was also a loss-of-function mutation reducing the binding of FGF8 it to its receptors. The current findings corroborate the value of zebrafish mutant models in the unraveling of the role of FGF8 in craniofacial development and the etiology of craniofacial malformations.

## MATERIALS AND METHODS

### Zebrafish breeding and husbandry

Zebrafish were raised and kept at 28°C under a 14 h light/10 h dark cycle with twice-a-day feeding at the Radboud University Zebrafish Facility. The mutant line *fgf8a^ti282^* (*ace*) was obtained from the European Zebrafish Resource Center (EZRC, Karlsruhe Institute of Technology, Germany). To obtain homo- and heterozygous mutant embryos, *fgf8a^ti282^* heterozygous adult carriers were pair-wise crossed. Every 2 weeks, breeding tanks were set up the day before mating, with males and females separated by a transparent wall. The following morning, the wall was removed and the water was changed for low-conductivity warm water of around 29°C to induce spawning. Eggs were collected approximately 60 min after spawning and transferred to petri dishes with E3 medium (5 mM NaCl, 0.17 mM KCl, 0.33 mM CaCl_2_, 0.33 mM MgSO_4_, 0.00001% Methylene Blue). Petri dishes with eggs were placed in an incubator at 28.5°C with a 14 h light/10 h dark cycle. E3 medium was refreshed at days 1, 4 and 5; any unfertilized eggs, dead embryos and post-hatching chorions were removed immediately. At 4 dpf, larvae were anaesthetized with 2-phenoxyethanol (1:750) and a tail clip biopsy was taken from each larvae as previously described (Wilkinson et al., 2013). Upon tail clipping, the larvae were individually housed in 12-well plates (Sigma-Aldrich, USA).

### Genotyping

DNA from the tail clip biopsy and from the post-*in situ* samples was extracted through incubation in 15 µl NaOH (50 mM) for 30 min at 95°C. Samples were vortexed twice. After incubation, samples were placed on ice for 10 min and 1.5 µl of 1 M Tris (pH=8) was added. The DNA concentration of the samples was measured spectrophotometrically at 260 nm wavelength using a NanoDrop™ (Thermo Fisher Scientific, Wilmington, USA) and adjusted to a final quantity of 30 ng. The DNA in each individual sample was amplified by a tetra-primer amplification refractory mutation (ARMS) PCR, with a not allele-specific (outer) pair and an allele-specific (inner) pair of primers. Primer sequences used were: forward in (A) 5′GGGAAACTGATTGGCACGA 3′; reverse in (G) 5′TCACAAAAGTGATGACTTTTTCACAGAC 3′; forward out 5′TTTGGGAGTCGAGTTCGAATTAA 3′; reverse out 5′TTTTTTTCCCTTTCTAGGTGGGA 3′. For each PCR reaction, 6 pmol of both inner primers and 1.5 pmol of both outer primers was used. PCR products were loaded on a 2% agarose gel (SeaKem LE Agarose, Lonza) and separated by electrophoresis. The three possible amplicons are a 213-bp non-allele specific, a 151-bp mutant (A) allele-specific, and a 109-bp wild-type (G) allele-specific fragment.

For genotyping after gene expression analysis a slightly different method was used. Here, 3 μl of cDNA of each individual sample was mixed with 17 μl QPCR mix and amplified by PCR using the following primers: forward; GGTGAGCCGTAGACTAATCCG and reverse; GTGTTGCCTGGTTTTGGAGC. The PCR products were loaded on a 2% agarose gel (SeaKem LE Agarose, Lonza) and separated by electrophoresis. The two possible amplicons are a 370 bp wild-type (G) fragment and a 263 bp mutant (A) fragment.

### Cartilage and bone staining

At 5 dpf, larvae identified as wild type or heterozygous for *fgf8a^ti282^* through tail-clip genotyping were grouped by genotype and stained in an Eppendorf tube for cartilage and bone based on the acid-free staining protocol by Walker and Kimmel (2007) with some modifications. Larvae were euthanized with 0.1% 2-phenoxyethanol (1:500) and fixated for 30 min in 2% paraformaldehyde. After washing with water and 80% ethanol, larvae were incubated in staining solution (0.02% Alcian Blue; Sigma-Aldrich, containing 40 mM of MgCl_2_) for 1.5 h to stain cartilage. Larvae were washed in ethanol and cleared through bleaching in peroxide (0.8% KOH, 1.1% H_2_O_2_, 0.2% Triton) followed by two washing steps: first in 0.2% Triton and then in a saturated sodium tetraborate solution. This was then followed by trypsin digestion (1 mg/ml) for 15 min and a wash in 0.2% Triton. Subsequently, bone was stained overnight with 0.003% Alizarin Red (Sigma-Aldrich). Finally, the larvae were cleared in graded series of glycerol (25%, 50% and 75%) and stored in 100% glycerol for imaging.

Homozygous *fgf8a^ti282^* larvae could be phenotypically identified at 30 hpf by an enlarged tectum and lack of a mid-hindbrain boundary (Brand et al., 1996). Because homozygote larvae were very fragile due to edema, they were stained individually in a 48-well plate. Larvae were euthanized with 0.1% 2-phenoxyethanol and fixated for 30 min in 2% paraformaldehyde. After washing with 100 mM Tris/40 mM MgCl_2_ (pH 7.5)_,_ larvae were stained with Alcian Blue as described. Larvae were washed for 5 min in a series of 80% ethanol [100 mM Tris/40 mM MgCl_2_ (pH 7.5)] and 50 and 25% ethanol [100 mM Tris (pH 7.5)] and bleached in a H_2_O_2_ solution (3% H_2_O_2,_/0.5% KOH), followed by two wash steps with 25% glycerol/0.1% KOH for 10 min. Subsequently, bone was stained overnight with 0.01% Alizarin Red (pH 7.5, Sigma-Aldrich). The next day, the larvae were washed twice with 50% glycerol/0.1% KOH for 10 min and stored in 100% glycerol for imaging. Following imaging, whole larvae were washed in PBS and genotyped according to the described procedure.

### Zebrafish imaging

Larvae stained for cartilage and bone stored in 100% glycerol were loaded into round borosilicate glass capillaries (CV6084-100, Vitrocom, USA), which were placed inside square borosilicate capillaries (CV8290-100, Vitrocom, USA) also filled with 100% glycerol. The capillaries were placed in a sample holder with an axial rotating system (adapted from Bruns et al., 2015) and images were acquired from dorsal, ventral and lateral sides of the larvae with a binocular microscope (Leica DMRE) using Leica Application Suite (LAS 3.3, Leica).

The embryos stained with *in situ* probes were cleared in a 2:1 mixture of benzyl benzoate (Merck) and benzyl alcohol (Merck) during imaging. Images were acquired with a binocular microscope (Leica DMRE) using Leica Application Suite (LAS 3.3, Leica).

### Bone and cartilage analysis

**Fig. 7.**
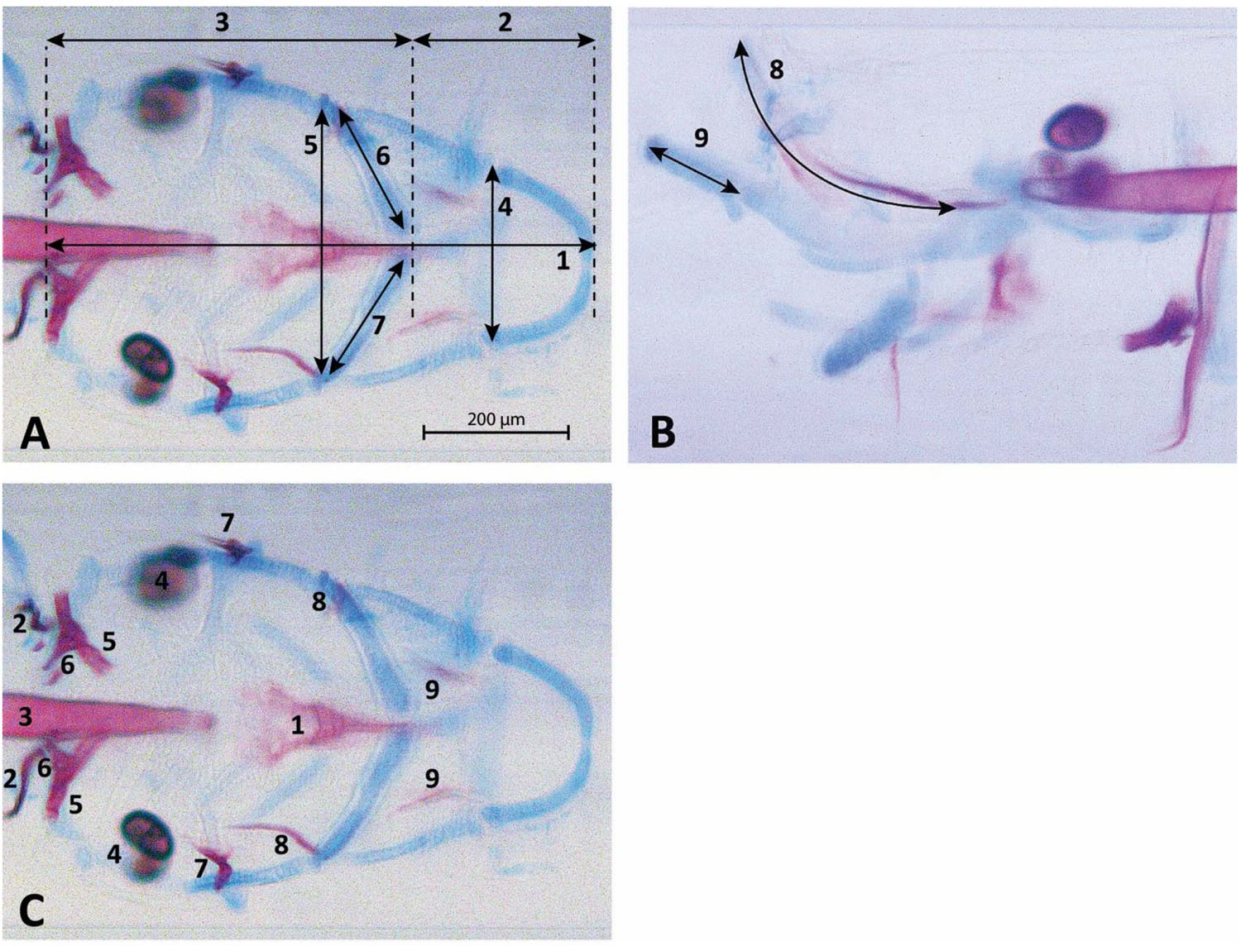
**5 dpf zebrafish larva (wild type) stained for cartilage (blue) and bone (red).** A total of nine parameters for cartilage were assessed as shown in panels A (ventral view) and B (lateral view): (1) total head length, (2) length ceratohyal to anterior end of the head, (3) length ceratohyal to posterior end of the head, (4) width at Meckel's cartilage and palatoquadrate joint, (5) width at ceratohyal and palatoquadrate joint, (6) length of superior ceratohyal, (7) length of inferior ceratohyal, (8) length of ethmoid plate, (9) lateral length of Meckel's cartilage. (C) The nine bone parameters scored for presence or absence (ventral view): (1) parasphenoid, (2) cleithrum, (3) notochordal sheath, (4) otoliths (four in total), (5) ceratobranchial 5, (6) teeth (on ceratobranchial 5), (7) opercles, (8) branchiostegal ray 1, (9) entopterygoid bone.

Pictures were imported in FIJI (Schindelin et al., 2012) and the following craniofacial cartilage parameters were measured: (1) total head length, (2) length ceratohyal to anterior end of the head, (3) length ceratohyal to posterior end of the head, (4) width at Meckel's cartilage and palatoquadrate joint, (5) width between ceratohyal and palatoquadrate joint, (6) length of superior ceratohyal, (7) length of inferior ceratohyal, (8) length of ethmoid plate, (9) length of Meckel's cartilage from the lateral side. The straight-line tool was used to measure most parameters, but for curved elements the segmented-line tool was used. For mineralized tissue stained by Alizarin Red, nine elements were scored for presence or absence of mineralization: (1) parasphenoid, (2) cleithrum, (3) notochordal sheath, (4) otoliths (all four present or not), (5) teeth (on ceratobranchial 5), (6) ceratobranchial 5, (7) opercles, (8) branchiostegal ray 1, (9) entopterygoid bone. All parameters are depicted in Fig. 7.

### Gene expression analysis

Another 96 larvae were randomly selected from a pool of offspring from heterozygous adults to ensure a blind procedure and euthanized with 0.1% 2-phenoxyethanol. Upon euthanasia, individual larvae were transferred to 2-ml Eppendorf tubes containing a plastic grinding ball and the total RNA of each sample was isolated. To this end, the larvae were homogenized in 400 μl Trizol reagent (Invitrogen, Carlsbad, USA) using a grinding mill for 20 s at 20 Hz. Samples were incubated at room temperature for 5 min and 80 μl chloroform was then added. Tubes were shaken for 15 s, followed by incubation at room temperature for 2 min. Samples were centrifuged at 18,000 ***g*** for 10 min in a cooled centrifuge (4°C), and 200 μl of the aqueous phase was transferred into a new tube. Isopropanol (200 μl) was added and mixed by inversion of the tube. The solution was stored at −20°C for 1 h and then centrifuged for 15 min at 18,000 ***g*** in a cooled centrifuge. The pellet was washed with 75% ethanol, then air-dried for 10 min at room temperature and dissolved in 100μl DEPC-treated water. 10 μl 3M sodium acetate (pH=5.4) and 250 μl 100% ethanol were added. Samples were stored overnight at −20°C. The following day, the samples were centrifuged for 15 min at 18,000 ***g***, the supernatant was decanted and the pellet was washed and then dissolved in 10 μl DEPC-treated water. The RNA concentration and purity of the samples were determined using a NanoDrop™ spectrophotometer at 260 nm wavelength (Thermo Fisher Scientific).

The thus isolated RNA was treated with DNase to remove traces of genomic DNA. RNA (200 ng) was transferred into a PCR strip and DEPC-treated water was added to a total volume of 8 μl. 2 μl DNase mix [1 μl 10X DNase I reaction buffer and 1 μl (1 U/μl) amplification grade DNase I (both from Invitrogen)] was added and the solution was incubated for 15 min at room temperature. After incubation, 1 μl 25 mM EDTA was added to stop the DNase reaction and the reaction mix was incubated for 10 min at 65°C and stored on ice. Samples were used to synthesize cDNA by adding 1 μl random primers (250 ng/μl), 1 μl 10 mM dNTP mix, 4 μl 5X 1st strand buffer, 1 μl 0.1 M DTT, 1 μl RNase inhibitor (10 U/μl), 0.5 μl Superscript II (reverse transcriptase 200 U/μl) (all from Invitrogen) and 0.5 μl DEPC-treated water. The resulting mix was incubated for 10 min at 25°C for annealing of the primers and then for 50 min at 42°C for reverse transcription. Enzymes were inactivated by incubation at 70°C for 15 min. Finally, the samples were diluted five times to serve as template in the qPCR reaction.

A real-time qPCR was carried out for each gene of interest. For each qPCR reaction, 4 μl of cDNA was mixed with 16 μl PCR mix (containing 10 μl SYBR green mix (2X) (Bio-Rad, Hercules, USA), 0.7 μl of each gene-specific primer (10 μM and 4.6 μl water). Primers used are listed in Table 1. The qPCR reaction (3 min 95°C, 40 cycles of 15 s 95°C and 1 min 60°C) was performed using a CFX 96 (Bio-Rad) qPCR machine. Threshold cycles (Ct values) were assessed and relative expression was calculated based on a normalization index of two reference genes: elongation factor alpha (*elf1a*) and ribosomal protein L13 (*rpl13*) (Vandesompele et al., 2002).

**Table 1.**
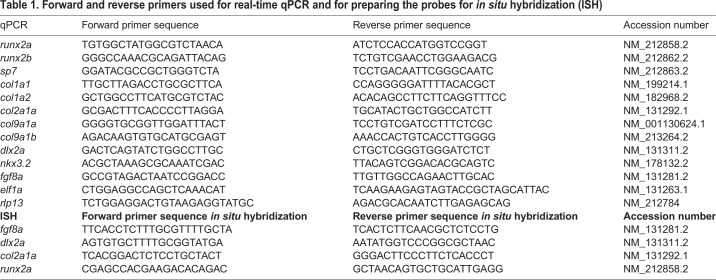
**Forward and reverse primers used for real-time qPCR and for preparing the probes for *in situ* hybridization (ISH)**

### *In situ* hybridization

Wholemount *in situ* hybridization was performed essentially as previously described (Thisse and Thisse, 2008). In short, primer sequences to generate *in situ* probes for *col2a1a*, *dlx2a*, *fgf8a* and *runx2a*, are listed in Table 1. Probe sequences were amplified by PCR and cloned in pGEM^®^-T Vector (Promega). Positive clones were confirmed by sequencing. Plasmids were linearized with *Notl* and *Ncol* (Thermo Fisher Scientific) and purified on column using GeneJET PCR purification kit (Thermo Fisher Scientific). Synthesis of digoxigenin (DIG)-labeled probes was performed using DIG labeling kit Sp6/T7 (Roche). An additional purification was performed using RNeasy plus micro kit (Qiagen).

8 hpf and 24 hpf-old embryos and larvae from crosses between heterozygous fish were fixed in 4% paraformaldehyde (PFA) for 12 h. Chorions were removed mechanically in PBS followed by dehydration in methanol. Upon use, samples were rehydrated in PBT (PBS+0.1% Tween20; Sigma-Aldrich) under gentle agitation. No proteinase K treatment was performed. Samples were pre-hybridized at 70°C for 2 h in hybridization mix (HM) consisting of 50% formamide (VWR), 5 x saline-sodium citrate buffer (SSC), 50 µg/ml heparin (Sigma-Aldrich), 500 µg/ml tRNA (Sigma-Aldrich), 0.1% Tween20 and 9,2 mM citric acid. Next, hybridization with DIG-labeled anti-sense probes was performed overnight at 70°C in HM (∼100 ng/ml probe). After hybridization, samples were washed in gradients of HM (without heparin and tRNA) and 2× SSC and transferred to 0.2× SCC at 70°C. After successive changes to PBT, pre-incubation was performed using 2% normal calf serum (NCS) (HyClone) in PBT. Samples were incubated with anti-DIG AP fragments (Roche) (1:2000) in 2% NCS overnight at 4°C. After six washes in PBT, samples were rinsed twice in alkaline phosphatase buffer consisting of 100 mM Tris (pH=9.5), 50 mM MgCl_2_, 100 mM NaCl and 0.1% Tween 20. Staining was achieved using Nitro Blue Tetrazolium (NBT)/5-Bromo-4-chloro-3-indolyl phosphate (BCIP) (Roche) in alkaline phosphatase buffer. The staining reaction was stopped by adding PBS (pH=5.5) supplemented with 1 mM EDTA. Subsequently, larvae were imaged as described in the imaging section. Following imaging, larvae were genotyped as described in the section on genotyping.

### Statistical analysis

All statistical analyses were performed using GraphPad Prism (version 5.03). Data were first examined for normality with the D'Agostino-Pearson normality test. For normally distributed parameters (one to three), data were statistically compared using a one-way ANOVA (with parameters one to three as repeated measures; genotype as independent factor) followed by a post-hoc Tukey HSD test. The not normally distributed data (parameters four to nine) were compared using a Kruskal–Wallis test (with parameters four to nine as repeated measures; genotype as independent factor) followed by a post-hoc Dunn's Multiple Comparison test. A Bonferroni correction was applied resulting in a significance limit of 0.05/9=*P*<0.0056. The data of the mineralized elements were statistically compared to each other with a Chi-Square test. Also here a Bonferroni correction was applied, resulting in a significance limit of 0.05/9=*P*<0.0056. As some of the data for gene expression were not normally distributed among groups, a Kruskal–Wallis test (with gene expression level as repeated measure; genotype as independent factor) was performed, followed by Dunn's multiple comparison test.
